# Prevalence and influencing factors of malnutrition in stroke patients with bulbar paralysis: a cross-sectional study in China

**DOI:** 10.3389/fnut.2024.1392217

**Published:** 2024-04-17

**Authors:** Hongji Zeng, Lianlian Liu, Ang Cai, Weijia Zhao, Yahui Liu, Liugen Wang, Heping Li, Xi Zeng

**Affiliations:** ^1^School of Public Health, Zhengzhou University, Zhengzhou, China; ^2^Department of Rehabilitation Medicine, The First Affiliated Hospital of Zhengzhou University, Zhengzhou, China; ^3^NHC Key Laboratory of Prevention and Treatment of Cerebrovascular Diseases, Zhengzhou, China

**Keywords:** malnutrition, stroke, bulbar paralysis, influencing factor, nutritional status, intermittent oro-esophageal tube

## Abstract

**Background:**

Although malnutrition has been shown to influence the clinical outcomes of Stroke Patients with Bulbar Paralysis (SPBP), the prevalence and influencing factors have yet to be uncovered.

**Objective:**

This study aims to assess the current prevalence and factors associated with malnutrition in SPBP.

**Methods:**

A multicenter cross-sectional investigation was conducted among SPBP in China from 2019 to 2021. Information was collected on basic information, health condition, diagnosis, treatment, neurological function, activities of daily living, swallowing function, and nutritional status. A multivariable logistic regression model was used to identify the factors that influenced nutritional status. ROC analysis was used to assess the predictive value of each independent influencing factor and the logit model.

**Results:**

In total, 774 SPBP were enrolled, and the prevalence of malnutrition was 60.59%. Pulmonary infection [aOR:2.849, 95%CI: (1.426, 5.691)], hemoglobin [aOR: 0.932, 95%CI: (0.875, 0.982)], serum albumin [aOR: 0.904, 95%CI: (0.871, 0.938)], total protein [aOR: 0.891, 95%CI: (0.819, 0.969)], prealbumin [aOR: 0.962, 95%CI: (0.932, 0.993)], and National Institute of Health Stroke Scale (NIHSS) scores [aOR: 1.228, 95%CI: (1.054, 1.431)] were independent factors associated with malnutrition in SPBP. ROC analysis revealed that the logit model had the best predictive value [area under the curve: 0.874, 95% CI: (0.812, 0.936); specificity: 83.4%; sensitivity: 79.3%; *p* < 0.05]. Subgroup analysis showed that the nutritional status in dysphagic SPBP was additionally influenced by swallowing function and nutrition support mode.

**Conclusion:**

The prevalence of malnutrition in SPBP was 60.59%. Pulmonary infection, hemoglobin level, and NIHSS score were the independent factors associated with malnutrition. Swallowing function and nutrition support mode were the factors associated with malnutrition in dysphagic SPBP.

## 1 Introduction

Stroke is a prevalent neurological disorder typically caused by abrupt rupture or obstruction of blood vessels in the brain, resulting in cerebral tissue damage (1). It is the second leading cause of death worldwide and can result in permanent neurological injury, serious complications, and even mortality (2, 3). According to the Global Burden of Disease Study, from 2005 to 2019, while there were fluctuations in the prevalence of hemorrhagic and ischemic strokes in China, the overall stroke prevalence has been on the rise (4). Furthermore, during the same period, the prevalence of stroke in China has persistently exceeded the global average and the averages of developed countries such as the United Kingdom, the United States, and Japan (5).

Stroke-related bulbar paralysis is a distinctive subtype of stroke, characterized by lesions in the medulla oblongata (6). It results from damage to the lower motor neurons of the medulla oblongata, including the nucleus ambiguus, hypoglossal nucleus or their associated lower motor neurons (7). In addition to stroke typical symptoms, individuals with bulbar paralysis often experience difficulties in speech and swallowing (8). Previous research has established a significant association between stroke and malnutrition (9). Specifically, stroke substantially increases feelings of fatigue, impairs digestive function, and leads to negative psychological states, ultimately resulting in individual malnutrition (10, 11). Moreover, dysphagia in Stroke Patients with Bulbar Paralysis (SPBP) is typically severe (12). Dysphagia-related complications including aspiration and reflux can cause additional consumption and loss of nutrients, thereby increasing the risk of malnutrition (13). In turn, malnutrition prolongs the duration of illness, impedes the recovery process, and escalates healthcare costs, hence establishing a vicious circle that burdens both the patients and their families (14). Given the significant impact of stroke-related bulbar paralysis on swallowing function, it holds clinical significance to consider the nutritional status of affected individuals.

Although the nutritional status of stroke patients has received increasing attention, current research primarily focuses on the impact of different interventions and factors on the nutritional status of stroke survivors (15). Moreover, the pathological and clinical presentations of SPBP differ from those of typical stroke survivors. However, there is a relative dearth of research specifically targeting this population (16). Based on this, we speculated that there may be certain differences in the prevalence and influencing factors of malnutrition. This lack of understanding and specific identification of factors associated with malnutrition in these patients may potentially hinder efforts to prevent and manage malnutrition in clinical practice. Therefore, this study was carried out with two objectives. First, we aimed to investigate the prevalence of malnutrition in SPBP. Second, we identified the factors associated with malnutrition in this population.

## 2 Method

### 2.1 Procedures and study participants

This was a multicenter cross-sectional study. On July 10, 2018, we conducted a preliminary on-site investigation at the First Affiliated Hospital of Zhengzhou University to estimate the sample size required for the formal investigation. Additionally, although most of the questionnaire contents were based on medical records, some aspects were affected by subjective factors. Therefore, we assessed the reliability and validity of the questionnaire through the preliminary survey.

The preliminary survey lasted for 6 months, and it is worth noting that the First Affiliated Hospital of Zhengzhou University has multiple rehabilitation departments. We initiated the preliminary survey simultaneously in all these departments. The selection criteria for the formal investigation and the preliminary survey were the same. The subjects of the preliminary survey were stroke patients, and data from those with bulbar paralysis were used for sample size estimation. After excluding invalid questionnaires, 379 participants were included in the preliminary survey. Through testing, we obtained reliability (Cronbach’s alpha = 0.916) and validity (KMO = 0.951) of the questionnaire. Among the 379 stroke patients, there were 143 cases (37.73%) diagnosed with medullary palsy and the malnutrition rate in these 143 cases was 43.36%.

Based on these results, we estimated the sample size, assuming a type I error probability of α = 0.05, *p* = 43.36%, and a margin of error of 0.05. The formula was as follows:


$$n={\left(Z_{/2}\right)}^{2*}\;\left[p\left(1-p\right)\right]/{\left(d\right)}^{2}\approx 377$$

Based on an estimated questionnaire efficiency of 80%, we initially estimated that at least 453 SPBP were required to support this study. From June 1, 2019, we conducted subsequent cross-sectional on-site investigations in five hospitals across China. However, due to policy restrictions in China, we were unable to obtain the cooperation of many hospitals when conducting stratified random sampling. Therefore, we conducted stratified random sampling among the cooperative hospitals of the Dysphagia Research Institute of Zhengzhou University, as follows:

Mainland China was divided into five major regions: South China, North China, East China, West China, and Central China. In each region, we randomly selected a tertiary hospital collaborating with the Dysphagia Research Institute of Zhengzhou University and conducted on-site investigations every 15 days. The invited participants were not informed of the topic of the survey before providing preliminary consent. To ensure a sufficient sample size for estimating the prevalence of malnutrition among SPBP, we dynamically conducted questionnaire quality control and partial data recording throughout the study to monitor sample saturation. In this study, saturation refers to the point at which the sample size reaches a specific level, where the prevalence of malnutrition no longer changes significantly with increasing sample size. The sample began to saturate when the number of SPBP reached 613. Subsequently, on July 9, 2021, we completed the investigation when the number of SPBP reached 827, resulting in a total of 1762 stroke patients. After quality control, 774 SPBP and 1,631 cases of stroke patients were enrolled, with effective rates of 93.59 and 92.57%, respectively. All eligible participants were investigated only once, even if they were hospitalized in subsequent rounds.

The inclusion criteria were: (1) Age ≥ 18 years. (2) Meeting the diagnostic criteria for stroke established by the Neurology Science of the Chinese Medical Association (17), confirmed through brain CT or MRI. (3) First-onset stroke. (4) Stable vital signs. (5) Transferred to the Department of Rehabilitation Medicine within 15 days of onset. (6) Patients who received rehabilitation treatment and care including intracranial pressure reduction, elimination of oxygen free radicals, and antiplatelet aggregation drugs. (7) Conscious and able to complete the investigation. (8) Patients who adhered to the same nutritional support mode for at least seven consecutive days. (9) Not receiving parenteral nutrition until the time of survey.

The exclusion criteria were: (1) Combined major organ failure such as heart, lung, liver, and kidney. (2) Combined malignant tumors, blood disorders, depression, and anxiety. (3) Combined autoimmune diseases. (4) Combined chronic debilitating diseases prior to stroke.

Specifically, patients with stroke occurring in the medulla oblongata were regarded as having bulbar paralysis.

The study was conducted in accordance with the Declaration of Helsinki and relevant guidelines, and approved by the Ethics Committee of the First Affiliated Hospital of Zhengzhou University (2021-KY-0609-003). Written informed consent was obtained from each patient prior to enrollment in the study.

### 2.2 Assessment

#### 2.2.1 Basic information

Basic information was collected, including age (years), body mass index (BMI, kg/m^2^), gender (Male/Female), hypertension (Yes/No), diabetes (Yes/No), coronary heart disease (Yes/No), smoking (Yes/No), alcohol intake (Yes/No), pulmonary infections (Yes/No), location of medullary injury (Left side/Right side/Bilateral), nutrition support mode (Intermittent oro-esophageal tube/Nasogastric tube/Oral intake), total cholesterol (mmol/L), lymphocyte count (10^9^/L), hemoglobin (g/L), serum albumin (g/L), total protein (g/L).

Data on personal information and illness were based on medical records. Specifically, the patients BMI was assessed with the combination of height and body weight, where BMI < 18.5 kg/m^2^ was classified as underweight, BMI ranging from 18.5 to 23.9 kg/m^2^ was considered normal weight, BMI between 24.0 and 27.9 kg/m^2^ was categorized as overweight, and BMI ≥ 28.0 kg/m^2^ was classified as obesity (18). A patient who had smoked at least one cigarette per week for at least 6 months prior to the day of the investigation was regarded as smoking-Yes based on self-report (3). A patient who had consumed alcohol for at least 10 mL per week for at least 6 months was regarded as alcohol intake-Yes based on self-report (3). A patient who was diagnosed with hypertension, diabetes, or coronary heart disease within 5 years was regarded as Yes. Pulmonary infections were examined during hospitalization. The location of the medullary injury was based on the imaging materials. Intermittent oro-esophageal tube (IOE) and nasogastric tube (NGT) are the main enteral nutrition support modes in China (19). IOE refers to a feeding tube made of silicone that is intermittently inserted into the patient’s mouth to reach the upper end of the esophagus. During feeding, positive pressure from the oral cavity, negative pressure from the stomach, and swallowing movements in the throat help the food enter the stomach. After feeding, the tube was immediately removed (20). If there were existing data on total cholesterol, lymphocyte count, and hemoglobin within 3 days before the investigation, the data would be used. If not, the patient was instructed to undergo a routine blood test including these indicators within the following 3 days, and the data were subsequently recorded.

#### 2.2.2 Neurological function

Neurological function was assessed using the National Institutes of Health Stroke Scale (NIHSS), which is commonly used in stroke patients. The NIHSS includes 11 items that cover the most common neurological functions in stroke patients, such as level of consciousness, eye movement, facial muscle activity, upper and lower limb motor abilities, limb coordination, sensory function, and language. The personnel involved in this assessment received medical education and detailed training in NIHSS assessment. The maximum score on the scale is 42, with a higher score indicating poorer neurological function (21).

#### 2.2.3 Ability of daily living

The patient’s activities of daily living (ADL) were assessed using the Modified Barthel Index (MBI). Through observation and questioning of the patient, this scale can be used to evaluate independence in ADL, including eating, bathing, dressing, moving, using the toilet, and controlling the bowel and bladder. Each item is scored from 0 to 5, representing the level of independence in that activity. The total scores ranged from 0 to 100, with higher scores indicating better ADL. The staff involved received detailed training during the MBI assessment (22).

#### 2.2.4 Swallowing function

Swallowing function was assessed using the Functional Oral Intake Scale (FOIS), which is widely used in clinical practice and research to evaluate a patient’s ability to consume food and drink orally. The FOIS consists of seven levels, each of which describes specific oral intake states and dietary types. The higher the level, the better is the swallowing function. A FOIS level ≤ 3 or below is considered indicative of dysphagia (23). We assigned a professional therapist to conduct FOIS assessments on each hospital’s team to minimize subjective bias.

#### 2.2.5 Nutritional status

Relevant guidelines suggest that the diagnosis of malnutrition should be based on screening for malnutrition (24). Screening was performed using the Nutritional Risk Screening 2002 (NRS-2002) criteria (25). An NRS-2002 score > 3 indicated a risk of malnutrition. For patients at this risk, the Global Leadership Initiative on Malnutrition (GLIM) criteria 2016 were used for diagnosis (26). The diagnostic criteria included three phenotypic criteria and two etiological criteria. Malnutrition can be diagnosed when a patient meets at least each one of the two sets of criteria. After the assessment, the participants were divided into malnutrition and normal nutrition groups accordingly.

### 2.3 Statistical analysis

Normally distributed and homoscedastic continuous variables were presented as mean and deviation (x ± s), and group comparisons were conducted using an independent samples t-test. Non-normally distributed continuous variables were presented as median and quartile [M, (P_25_, P_75_)], and group comparisons were performed using the Mann–Whitney *U* test. Categorical variables were presented as number of cases and frequency (*n*, %), and group comparisons were conducted using the chi-square test. Multiple logistic regression analysis was used to identify the risk factors for malnutrition in SPBP and establish a predictive model. Collinearity testing was performed to ensure that the variables included in the analysis were independent of each other. Receiver operating characteristic (ROC) analysis was used to evaluate the predictive value of the model. Subsequently, the dysphagic SPBP were included in the subgroup analysis for comparison of influencing factors among the dysphagic versus non-dysphagic participants. A *p*-value<0.05 was regarded as statistically significant. SPSS 21.0 Statistical was used for data analysis.

## 3 Result

### 3.1 Comparison of the prevalence of malnutrition

Totally, 774 cases of SPBP from 1,631 cases of stroke patients were enrolled. Among them, 469 cases of SPBP (60.59%) and 832 cases of all the participants with stroke (51.01%) were, respectively, identified as malnutrition, with a significant difference. In addition, there were statistically significant differences (*p* < 0.05) in BMI, and BMI classification between the two groups, as shown in Table 1.

**Table 1:** Comparison between SPBP and all stroke patients.

Variables	SPBP (*n* = 774)	Stroke patients (*n* = 1,631)	*Z/X^2^*	*P*
Malnutrition (*n*, %)			19.410	<0.001***
Yes	469 (60.59)	832 (51.01)		
No	305 (39.41)	799 (48.99)		
BMI [kg/m^2^, M, (P_25_, P_75_)]	21.91 (19.67, 23.94)	22.75 (20.54, 24.32)	3.605	<0.001***
BMI classification (n, %)			15.572	0.001**
Underweight	126 (37.08)	212 (24.16)		
Normal weight	459 (26.79)	923 (56.59)		
Overweight	153 (14.68)	362 (28.72)		
Obesity	36 (21.45)	134 (11.13)		

*Z*: Mann–Whitney U test. *X*^2^: chi-square test.^*^p < 0.05, ^**^p < 0.01, ^***^p < 0.001.

### 3.2 Comparison between the SPBP

According to the nutritional status, the SPBP were divided into a normal nutrition group [*n* = 305 (39.41%)] and a malnutrition group [*n* = 469 (60.59%)]. There were no statistically significant differences in gender, hypertension, diabetes, coronary heart disease, smoking, alcohol intake, location of medullary injury, nutrition support mode, total cholesterol, lymphocyte count, and swallowing function between the two groups (*p* > 0.05). However, there were statistically significant differences between the groups in age, NIHSS score, hemoglobin, MBI, serum albumin, prealbumin, total protein, and pulmonary infection (*p* < 0.05), as shown in Table 2.

**Table 2:** Comparison of nutritional status in SPBP.

Variables	Malnutrition group (*n* = 469)	Normal nutrition group (*n* = 305)	*t/Z/X^2^*	*P*
Gender (*n*, %)			2.586	0.108
Male	392 (83.59)	241 (79.18)		
Female	77 (16.41)	64 (20.82)		
Coronary heart disease-Yes (*n*, %)	106 (22.61)	59 (19.34)	1.169	0.279
Diabetes-Yes (*n*, %)	181 (38.62)	132 (43.28)	1.685	0.194
Hypertension-Yes (*n*, %)	238 (50.74)	139 (45.57)	1.979	0.160
Smoking-Yes (*n*, %)	172 (36.69)	98 (32.13)	1.679	0.195
Alcohol intake-Yes (*n*, %)	92 (19.61)	75 (24.59)	2.702	0.100
Pulmonary infection-Yes (*n*, %)	319 (68.03)	177 (58.04)	8.004	0.005**
Location of medullary injury (*n*, %)			4.238	0.120
Left side	212 (45.18)	118 (38.69)		
Right side	190 (40.51)	146 (47.87)		
Bilateral	67 (14.31)	41 (13.44)		
Nutrition support mode (*n*, %)			1.927	0.382
IOE	181 (38.59)	133 (43.61)		
NGT	256 (54.59)	153 (50.16)		
Oral intake	32 (6.82)	19 (6.23)		
FOIS (*n*, %)			8.735	0.189
I	53 (11.31)	44 (14.43)		
II	103 (21.96)	79 (25.90)		
III	137 (29.21)	65 (21.31)		
IV	104 (22.17)	64 (20.98)		
V	44 (9.38)	37 (12.13)		
VI	15 (3.20)	9 (2.95)		
VII	13 (2.77)	7 (2.30)		
Age (years, x ± s)	60.34 ± 12.19	57.83 ± 14.62	2.585	0.009**
MBI (points, x ± s)	68.48 ± 13.79	71.52 ± 15.34	2.866	0.004**
Hemoglobin [g/L, M, (P_25_, P_75_)]	127.00 (118.50, 138.00)	137.00 (123.00, 145.00)	7.845	<0.001***
Total cholesterol [mmol/L, M, (P_25_, P_75_)]	3.37 (2.98, 4.02)	3.49 (2.92, 4.13)	0.395	0.693
Lymphocyte count [10^9^/L, M, (P_25_, P_75_)]	1.50 (1.10, 1.85)	1.55 (1.21, 1.95)	1.121	0.262
Prealbumin [mg/L, M, (P25, P75)]	204.80 (176.50, 228.80)	245.40 (219.30. 294.70)	16.068	<0.001***
Serum Albumin [g/L, M, (P_25_, P_75_)]	36.23 (35.14, 37.26)	40.55 (39.35, 42.59)	4.736	<0.001***
Total Protein (g/L, x ± s)	63.54 ± 8.84	65.82 ± 9.18	3.453	0.001**
NIHSS [points, M, (P_25_, P_75_)]	5.00 (3.00, 8.50)	2.00 (1.00, 4.00)	4.759	<0.001***

*t*, *t*-test; *Z*, Mann–Whitney *U* test. *X*^2^, chi-square test.^*^p < 0.05, ^**^p< 0.01, ***p < 0.001.

### 3.3 Logistic regression and ROC analysis

Variables with statistical differences (Table 1) were included in the multiple-factor logistic regression analysis. The results showed that pulmonary infection, NIHSS score, serum albumin, prealbumin, total protein, and hemoglobin were independent risk factors for malnutrition in SPBP, as shown in Tables 3, 4 for details.

**Table 3:** Collinearity diagnosis.

Variables	VIF	Tolerance
Age	1.083	0.923
Pulmonary infection	2.346	0.426
Hemoglobin	2.276	0.439
NIHSS	1.274	0.785
MBI	1.101	0.908
Serum albumin	1.942	0.515
Total protein	1.733	0.577
Prealbumin	2.464	0.406

**Table 4:** Multiple-factor logistic regression.

Variables	Outcomes	*B*	*S.E.*	*Walid X^2^*	aOR (95%CI)	*P*
Age		0.087	0.049	3.158	1.091 (0.991, 1.201)	0.076
Pulmonary infection						
	No	1.00 (ref)				
	Yes	1.047	0.353	8.796	2.849 (1.426, 5.691)	0.003**
Hemoglobin		–0.071	0.032	4.841	0.932 (0.875, 0.982)	0.027*
Serum Albumin		–0.101	0.019	28.207	0.904 (0.871, 0.938)	<0.001***
Total Protein		–0.115	0.043	7.203	0.891 (0.819, 0.969)	0.007**
Prealbumin		–0.039	0.016	5.863	0.962 (0.932, 0.993)	0.016*
NIHSS		0.205	0.078	6.917	1.228 (1.054, 1.431)	0.009**
MBI		−0.144	0.164	0.769	0.866 (0.628, 1.194)	0.381

^*^p < 0.05, ^**^p < 0.01, ^***^p < 0.001.All enrolled variables passed the univariate regression analysis with statistical significance. Adjusted variables: age, pulmonary infection, hemoglobin, serum albumin, total protein, prealbumin, NIHSS, and MBI.

### 3.4 ROC analysis of the logistic regression model

According to the logistic regression analysis results, the factors with significant differences in Table 4 were included in the equation to build a predictive model, which was obtained as follows:

Logit(p) = 4.178 + 1.047*Pulmonary infection−0.071* Hemoglobin−0.101* Serum Albumin−0.115*Total Protein−0.039* Prealbumin+0.205 *NIHSS.

**Figure 1:**
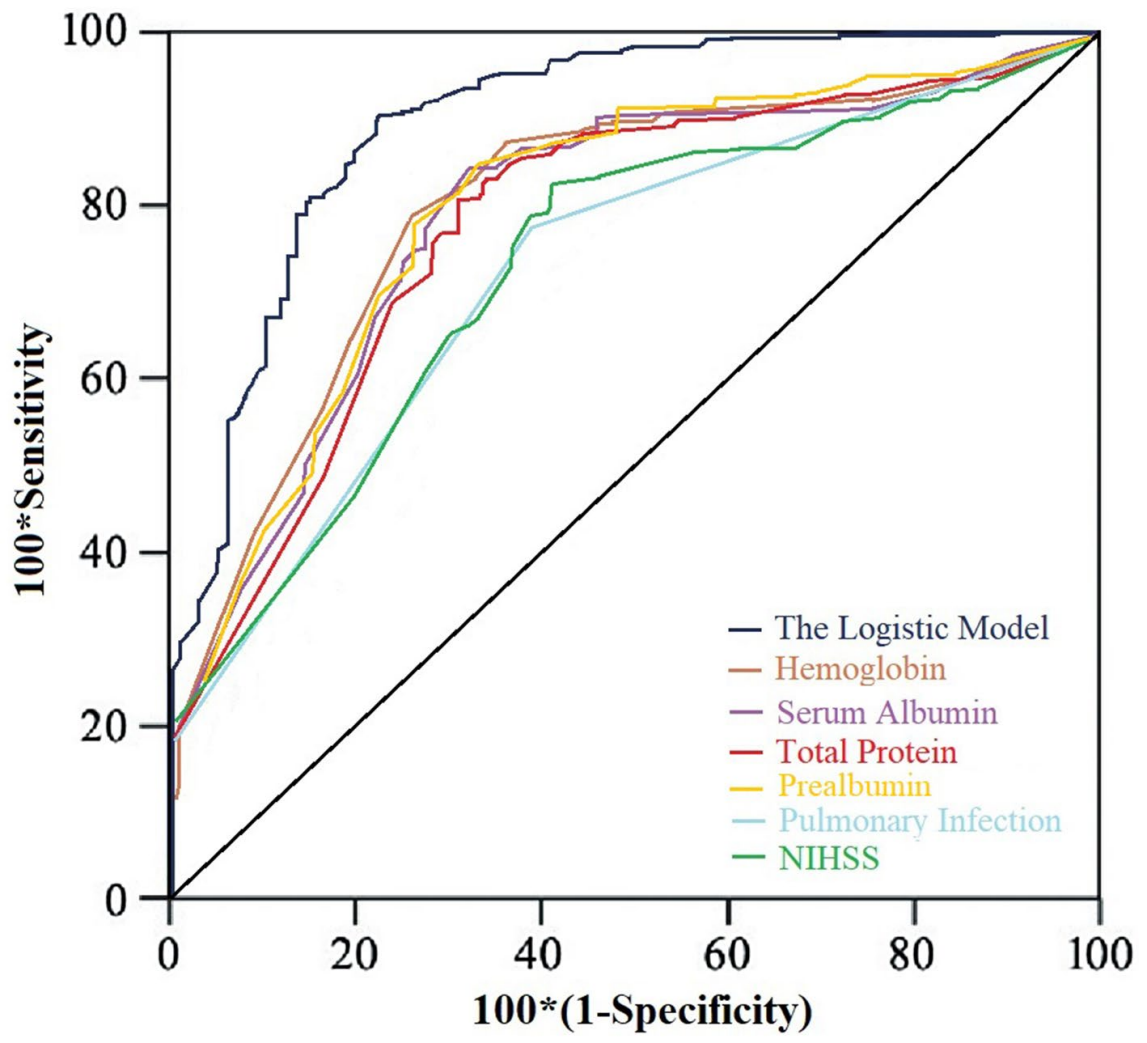
ROC curve.

The model’s goodness of fit was assessed using the Hosmer-Lemeshow test, which showed a chi-square value of 9.184 with a *p*-value of 0.421, indicating a good fit of the model. Moreover, ROC analysis showed that the model’s AUC was 0.874 (95% CI: 0.812, 0.936), with a specificity of 83.4% and a sensitivity of 79.3%. Additionally, the predictive performance of the model was significantly better than that of the individual indicators, as shown in Figure 1.

### 3.5 Subgroup analysis of dysphagic participants

The SPBP were classified into the dysphagia and non-dysphagia group according to their FOIS assessments. A FOIS level ≤ 3 was considered indicative of dysphagia. The dysphagic participants were included in the subgroup analysis. Univariate analysis showed that there were significant differences in pulmonary infection, nutrition support mode, FOIS, age, MBI, hemoglobin, prealbumin, serum albumin, total protein, and NIHSS, as shown in Table 5. A logistic regression was conducted with these variables (nutritional status as dependent variable). The results showed that, compared to the overall population of SPBP, the nutritional status of those with dysphagia was additionally influenced by the nutrition support mode and FOIS, as shown in Table 6.

**Table 5:** Comparison of nutritional status in the dysphagic participants.

Variables	Malnutrition group (*n* = 293)	Normal nutrition group (*n* = 188)	*t/Z/X^2^*	*P*
Gender (*n*, %)			0.963	0.326
Male	231 (78.96)	141 (75.00)		
Female	62 (21.04)	47 (25.00)		
Coronary heart disease-Yes (*n*, %)	59 (20.14)	38 (20.21)	0.941	0.332
Diabetes-Yes (*n*, %)	119 (40.61)	71 (37.77)	0.389	0.532
Hypertension-Yes (*n*, %)	168 (57.35)	97 (51.60)	1.526	0.216
Smoking-Yes (n, %)	126 (43.00)	69 (36.70)	1.886	0.169
Alcohol intake-Yes (*n*, %)	62 (21.18)	48 (25.53)	1.241	0.265
Pulmonary infection-Yes (*n*, %)	192 (65.55)	93 (49.47)	12.235	<0.001***
Location of medullary injury (*n*, %)			3.064	0.216
Left side	134 (45.74)	82 (43.62)		
Right side	142 (48.47)	87 (46.28)		
Bilateral	17 (5.79)	19 (10.11)		
Nutrition support mode (*n*, %)			10.065	0.006**
IOE	81 (38.98)	78 (41.49)		
NGT	199 (58.38)	102 (54.26)		
Oral intake	13 (2.63)	8 (4.25)		
FOIS (*n*, %)			7.080	0.029*
I	53 (18.09)	44 (23.40)		
II	103 (35.15)	79 (42.02)		
III	137 (46.76)	65 (34.57)		
Age (years, x ± s)	61.22 ± 9.81	58.37 ± 11.23	2.936	0.003**
MBI (points, x ± s)	64.74 ± 10.06	68.39 ± 9.28	3.979	<0.001***
Hemoglobin [g/L, M, (P_25_, P_75_)]	121.00 (109.50, 135.00)	132.00 (120.00, 139.00)	6.984	<0.001***
Total cholesterol (mmol/L, x ± s)	3.29 ± 0.93	3.38 ± 0.85	1.071	0.285
Lymphocyte count (10^9^/L, x ± s)	1.52 ± 0.41	1.48 ± 0.54	0.920	0.358
Prealbumin (mg/L, x ± s)	195.86 ± 49.31	243.19 ± 58.20	9.564	<0.001***
Serum Albumin (g/L, x ± s)	36.37 ± 4.17	39.82 ± 6.28	7.241	<0.001***
Total Protein (g/L, x ± s)	62.61 ± 9.81	64.94 ± 8.38	2.687	0.007**
NIHSS [points, M, (P_25_, P_75_)]	5.00 (3.00, 8.00)	2.00 (1.00, 4.00)	4.592	<0.001***

*t*, *t*-test; *Z*, Mann–Whitney *U* test. *X*^2^, chi-square test.^*^p < 0.05, ^**^p < 0.01, ^***^p < 0.001.

**Table 6:** Subgroup logistic regression.

Variables	Outcomes	*B*	*S.E.*	*Walid X^2^*	aOR (95%CI)	*P*
Age		0.120	0.077	2.446	1.128 (0.969, 1.311)	0.118
Pulmonary infection						
	No	1.00 (ref)				
	Yes	1.171	0.424	7.631	3.226 (1.405, 7.406)	0.006**
Nutrition support mode						
	IOE	1.00 (ref)				
	NGT	−0.553	0.271	4.170	0.575 (0.338, 0.978)	0.041*
	Oral intake	0.359	0.594	0.365	1.432 (0.447, 4.587)	0.546
FOIS						
	I	1.00 (ref)				
	II	0.554	0.358	2.399	1.741 (0.863, 3.512)	0.121
	III	0.797	0.324	6.045	2.218 (1.175, 2.459)	0.014*
Hemoglobin		−0.159	0.079	4.051	0.853 (0.731, 0.996)	0.044*
Serum albumin		−0.131	0.033	15.544	0.878 (0.823, 0.937)	<0.001***
Total protein		−0.171	0.084	4.134	0.843 (0.715, 0.994)	0.042**
Prealbumin		−0.039	0.039	5.061	0.916 (0.848, 0.988)	0.025*
NIHSS		0.353	0.143	6.109	1.424 (1.076, 1.885)	0.013*
MBI		−0.276	0.298	0.856	0.759 (0.423, 1.361)	0.355

^*^p < 0.05, ^**^p < 0.01, ^***^p < 0.001.All enrolled variables passed the univariate regression analysis with statistical significance. Adjusted variables: age, pulmonary infection, hemoglobin, serum albumin, total protein, prealbumin, NIHSS, FOIS, nutrition support mode, and MBI.

## 4 Discussion

The current study showed that the prevalence of malnutrition in SPBP was 60.59%, significantly higher than the overall prevalence of malnutrition in the stroke survivors enrolled (51.01%, *p* < 0.05). Possible reasons for this include the fact that the lesions in patients with true bulbar palsy are located in the medulla oblongata, which is the most basic center for regulating swallowing function (27). Therefore, SPBP typically experience severe dysphagia, manifested as difficulty in eating, inability to swallow food after chewing, choking on liquids, and in severe cases, inability to conduct oral intake, which can potentially lead to malnutrition. In addition, SPBP generally have poor sensitivity to swallowing rehabilitation training, which makes it difficult to improve their malnutrition status (28). Specifically, most of these patients experience severe dysphagia and hence rely on tube feeding. Although special enteral nutrients have been recommended and optional as a source of energy and nutrition (29), in clinical practice, most patients cannot afford it for a long time due to economic factors and instead choose to make their own blended diets (typically liquid food). Obviously, the energy density of homemade blended diets is generally too low to meet the target calorie requirements. Tube feeding restricts the overall volume of daily feeding, thereby leading to inadequate calorie intake. Therefore, attention from healthcare professionals to the nutritional status of SPBP is necessary to reverse this situation early.

In clinical practice, it has been observed that some SPBP have accepted nutritional support but are still at high risk of malnutrition. Therefore, this study investigated the risk factors for malnutrition in SPBP to help identify high-risk patients at an early stage. This study demonstrated a pneumonia prevalence of 60% in SPBP, consistent with a clinical study conducted in China (27). Logistic regression analysis revealed that pulmonary infection is an independent risk factor. The underlying mechanisms may include the following: due to dysphagia and feeding tube placement, the functionality of the patient’s oropharynx and esophageal sphincter is affected. This increases the risk of aspiration and reflux (30). In addition, these patients often exhibit respiratory muscle dysfunction, leading to weakened coughing and expectoration. This impairs the patient’s self-protective ability during aspiration and hence increases the risk of pulmonary infections (31). When pulmonary infection occurs, the patient is physically influenced by inflammatory mediators and is typically in a high metabolic state, leading to increased consumption and loss of protein and energy, which increases the risk of malnutrition (32). Furthermore, patients with pulmonary infections often experience poor appetite. The gastrointestinal reactions and inflammation caused by pneumonia may affect the absorption and utilization of nutrients, including proteins, fats, and carbohydrates. This can result in patients being unable to fully absorb and utilize these nutrients (33). Additionally, the use of antibiotics and other drugs can exacerbate gastrointestinal irritation, leading to a sustained decrease in protein and calorie intake, and hence further worsen the nutritional status. A study on the nutritional status of stroke patients revealed a significant correlation between pulmonary infections and malnutrition. However, the study did not investigate the differences across various subtypes of stroke. This weakness was partially addressed by the current study (34). Therefore, during the rehabilitation process of SPBP, it is essential to properly apply enteral nutrition, strengthen oral hygiene, and conduct early training for swallowing and respiratory function to reduce the risk of pulmonary infections.

Additionally, this study showed that for each 1-point increase in the NIHSS score, the probability of malnutrition in SPBP increased by approximately 1.228 times, which was similar to the findings reported by Cao (35). A study conducted in 2022 indicated that this association was not limited to the hospitalization period and remained significant after 3 months after onset (36). Generally, patients exhibit varying degrees of neurological deficits after stroke, including dysphagia, cognitive impairments, and decreased limb motor abilities. This can result in decreased self-feeding abilities, ADL, and appetite, thereby increasing the risk of malnutrition (37). In addition, neurological deficits can also affect patients’ ability to comply with rehabilitation training and impact the effectiveness of rehabilitation. This can slow down the recovery of various functions in patients, including eating and digestion, thereby affecting their nutritional status (38). Therefore, it is important to pay special attention to the nutritional status of patients with high NIHSS scores and provide timely and effective clinical nursing interventions. Furthermore, relevant guidelines and health education should be provided to the patients and their families.

The results of this study also showed that low hemoglobin, prealbumin, serum albumin and total protein were independent risk factors for malnutrition in SPBP, consisted with a previous study (39). Hemoglobin can physically reflect iron metabolism and protein status. Its main function is to carry oxygen and transport carbon dioxide to the lungs for elimination (40). Decreased hemoglobin levels can lead to local tissue hypoxia, resulting in decreased muscle strength, increased fatigue, and cognitive impairment in patients (41). Prolonged deficiency of hemoglobin, if left uncorrected, can further lead to anemia and even impairment of hematopoietic system function, increasing the risk of hypo-hemoglobinemia and malnutrition (42). Serum albumin is one of the most common proteins in human blood, synthesized by the liver. It is crucial in maintaining normal physiological status and overall health. Serum albumin is a small-molecule, water-soluble protein that primarily functions to maintain plasma osmotic pressure, regulate blood rheology, and transport various bioactive substances. Due to its high sensitivity to nutritional status and liver function during synthesis, serum albumin is routinely used as an important indicator for nutritional status (43). Total protein refers to the combined concentration of all proteins present in a biological sample, typically measured in units such as grams per deciliter in blood serum. It includes various types of proteins, such as albumin, globulins, and other plasma proteins. The measurement of total protein levels in the blood is often used as an indicator of overall protein status in the body. Total protein levels can be influenced by factors such as nutrition, liver function, kidney function, hydration status, and certain medical conditions. Therefore, analyzing total protein levels alongside specific protein fractions like albumin and globulins can provide valuable information about a person’s health status and nutritional adequacy (44). Prealbumin, also known as transthyretin, is another important marker used to assess nutritional status. It is a small molecule protein primarily synthesized by the liver, with a relatively short half-life of about 2–3 days. Prealbumin levels typically fluctuate in response to changes in protein and energy intake, making it a useful and sensitive indicator of nutritional status. Regarding this situation, healthcare professionals should pay timely attention to the hemoglobin levels of such patients and take active measures to maintain hemoglobin levels within the normal range to reduce the risk of malnutrition.

Dysphagia is a common symptom among SPBP (45). However, due to the cross-sectional design, some participants were in the late stages of rehabilitation therapy. Therefore, we conducted subgroup analysis to further explore the influencing factors of the nutritional status of the dysphagic participants. The results showed that swallowing function and nutrition support mode are influencing factors of nutritional status. Dysphagia can lead to difficulty in eating and tube feeding dependence (46). It can also cause patients to experience problems such as coughing and aspiration pneumonia during meals, which can lead to fear of eating and thus reduce their willingness to eat (47). Studies have shown a significant correlation between dysphagia and complications such as pneumonia (27). In clinical practice, various feeding tubes are routinely used to alleviate the impact of dysphagia. In China, NGT and IOE are the primary options. Compared to NGT, IOE does not affect the nasal, pharyngeal, and lower esophageal sphincter functions. This can not only reduce the risk of pneumonia and reflux but also provide a higher daily feeding volume, thus having advantages in improving nutritional status (27). However, in patients with FOIS >3, the nutrition support mode is no longer a significant influencing factor, which may be because the tube feeding dependence is low in these patients, and oral intake can partially facilitate nutritional status.

Based on the ROC analysis, the AUC of the logistic model was significantly higher than that for each single factor separately, indicating a higher predictive value for the logistic model. In fact, patients with low laboratory indicators, pneumonia, and high NIHSS scores are common in clinical practice. It is worth noting that all these variables are easy to obtain and do not require expensive tests, such as MRI. The models constructed based on these risk factors have significant value in predicting the risk of malnutrition in this patient population, thereby providing early warning information. Healthcare professionals should identify patients at risk of malnutrition among SPBP as early as possible and take proactive and effective measures to intervene in their risk factors to improve their nutritional status, accelerate functional recovery, and facilitate early return to their families and society.

By examining the saturation of samples, we obtained a reliable prevalence. Additionally, through a series of statistical analyses, we identified independent risk factors for malnutrition in this population, thereby providing a reference for clinical work. However, this study had some notable limitations. First, the study did not employ a perfect stratified random sampling method but instead conducted a random sampling within the scope of collaborating institutions. Moreover, due to limited resources, we only included one tertiary hospital in each region, which may have led to inadequate sample representativeness. In the future, efforts should be made to establish collaborations and include as many hospitals as possible. Second, the cross-sectional design makes it difficult to infer causal relationships between variables. Therefore, this study may have involved bidirectional causality. Future research should further explore the causal relationships between variables using longitudinal designs and random interventions.

## 5 Conclusion

In summary, in SPBP in the Department of Rehabilitation Medicine, the prevalence of malnutrition was 60.59%. Moreover, pulmonary infection, NIHSS scores, hemoglobin, prealbumin, serum albumin, and total protein were identified as independent risk factors for malnutrition in these patients. There were differences in influencing factors of malnutrition among dysphagic versus non-dysphagic SPBP.

## Data availability statement

The datasets presented in this article are not readily available because data that support the findings of this study are available on request from the corresponding author, ZX. The data are not publicly available due to the confidentiality policy of the First Affiliated Hospital of Zhengzhou University. Requests to access the datasets should be directed to bestzhj@gs.zzu.edu.cn.

## Ethics statement

The studies involving humans were approved by Ethics Committee of the First Affiliated Hospital of Zhengzhou University (2021-KY-0609-003). The studies were conducted in accordance with the local legislation and institutional requirements. The participants provided their written informed consent to participate in this study.

## Author contributions

HZ: Conceptualization, Data curation, Formal analysis, Investigation, Methodology, Software, Visualization, Writing – original draft, Writing – review & editing. LL: Conceptualization, Formal analysis, Investigation, Methodology, Visualization, Writing – original draft, Writing – review & editing. AC: Conceptualization, Data curation, Formal analysis, Investigation, Methodology, Visualization, Writing – review & editing. WZ: Conceptualization, Data curation, Formal analysis, Investigation, Software, Visualization, Writing – review & editing. YL: Conceptualization, Data curation, Investigation, Supervision, Visualization, Writing – review & editing. LW: Conceptualization, Data curation, Methodology, Project administration, Resources, Supervision, Writing – review & editing. HL: Conceptualization, Data curation, Investigation, Project administration, Resources, Supervision, Writing – review & editing. XZ: Conceptualization, Data curation, Funding acquisition, Investigation, Methodology, Project administration, Resources, Software, Supervision, Validation, Writing – review & editing.
